# Glucagon-like peptide-2 rescues memory impairments and neuropathological changes in a mouse model of dementia induced by the intracerebroventricular administration of streptozotocin

**DOI:** 10.1038/s41598-019-50167-3

**Published:** 2019-09-23

**Authors:** Sachie Sasaki-Hamada, Masaatsu Ikeda, Jun-Ichiro Oka

**Affiliations:** 1 Laboratory of Pharmacology, Faculty of Pharmaceutical Sciences, Tokyo University of Science, 2641 Yamazaki, Noda, Chiba, 278-8510 Japan; 2 Department of Physiology, School of Allied Health Sciences, Kitasato University, Sagamihara, 252-0373 Japan

**Keywords:** Alzheimer's disease, Molecular medicine

## Abstract

Glucagon-like peptide 2 (GLP-2) is derived from the proglucagon gene expressed in the intestines, pancreas and brain. Our previous study showed that GLP-2 improved lipopolysaccharide-induced memory impairments. The current study was designed to further investigated the potential of GLP-2 in memory impairment induced by intracerebroventricular administration of streptozotocin (ICV-STZ) in mice, which have been used as an animal model of sporadic Alzheimer’s disease (AD). STZ was administered on alternate days (Day-1 and Day-3) in order to induce dementia in male ddY mice. ICV-STZ-treated mice were administered GLP-2 (0.6 μg/mouse, ICV) for 5 days from 14 days after the first ICV administration of STZ. In these mice, we examined spatial working memory, the biochemical parameters of oxidative stress, or neurogenesis. The GLP-2 treatment restored spatial working memory in ICV-STZ-treated mice. ICV-STZ-treated mice showed markedly increased thiobarbituric acid reactive species (TBARS) and decreased glutathione (GSH) levels, and GLP-2 significantly restored these ICV-STZ-induced changes. GLP-2 also significantly restored neurogenesis in the subgranular zone of the dentate gyrus in ICV-STZ-treated mice. We herein demonstrated that GLP-2 significantly restored ICV-STZ-induced memory impairments as well as biochemical and histopathological alterations, and accordingly, propose that the memory restorative ability of GLP-2 is due to its potential to reduce oxidative stress.

## Introduction

Glucagon-like peptide-2 (GLP-2) is a 33-amino acid peptide derived from proglucagon in the gut and central nervous system (CNS)^1,2^. Centrally administered GLP-2 to rodents has been shown to suppress food intake^3,4^. Moreover, GLP-2 protected hippocampal neurons from glutamate excitotoxicity^4^, stimulated the proliferation of cultured rat cortical astrocytes^5^, and improved lipopolysaccharide (LPS)-induced memory impairments^6^. However, there has been no conclusive experimental evidence to explain the protective potential of GLP-2 in animal models of Alzheimer’s disease (AD).

AD is the most common neurodegenerative disease associated with dementia in the elderly. It has been estimated that 67.5 million individuals worldwide will have AD by 2030, and this number may reach 115.4 million by 2050^7^. The two types of AD are clinically indistinguishable. Familial AD has a genetic origin, whereas sporadic AD is the more common form and has an unknown etiology^8,9^. Neuroinflammation, head trauma, impaired glucose/energy metabolism, and diabetes are among the risk factors for sporadic AD^10^.

The intracerebroventricular administration of streptozotocin (ICV-STZ) at a low dosage to rodents does not alter peripheral glucose levels, and is used as an experimental model for sporadic AD^11,12^. Streptozotocin (STZ)-induced impairments in glucose/energy metabolism may be a potential source of oxidative stress, neuroinflammation, neuronal cell death, and cholinergic damage^12–14^. ICV-STZ has been shown to induce spatial learning impairments in the Morris water maze (MWM) test and tau phosphorylation in the rodent brain, which had a sporadic AD-like pathology^15^. Previous studies reported dysfunctions in adult neurogenesis in the ICV-STZ model^16,17^ and human AD^18–20^. In the present study, we investigated the effects of GLP-2 on spatial learning memory, oxidative stress, and neurogenesis using ICV-STZ-induced AD mice.

## Materials and Methods

### Animals

We purchased five-week-old male ddY mice (Japan SLC, Shizuoka, Japan), used them during the age of six weeks, and attempted to minimize the number of animals used and their suffering. All animals were kept in a controlled environment with a 12:12-h light schedule, temperature (23 °C), and relative humidity (55 ± 5%), and were provided *ad libitum* access to food and water. All experiments were performed according to internationally followed ethical standards and approved by the research ethics committee of Tokyo University of Science, which was conducted according to the guidelines of the National Institute of Health and Japan Neuroscience Society.

### Drugs and chemicals

All drug solutions were freshly prepared before use. STZ and 5, 5′-dithiobis (2-nitrobenzoic acid) (DNTB) were from Sigma-Aldrich (St. Louis, MO, USA). GLP-2 was from Peptide Inc. (Osaka, Japan). All other chemicals were from Wako Pure Chemical Industries (Osaka, Japan). STZ and GLP-2 were dissolved in phosphate-buffered saline (PBS).

### Study design and animal treatments

**Figure 1:**
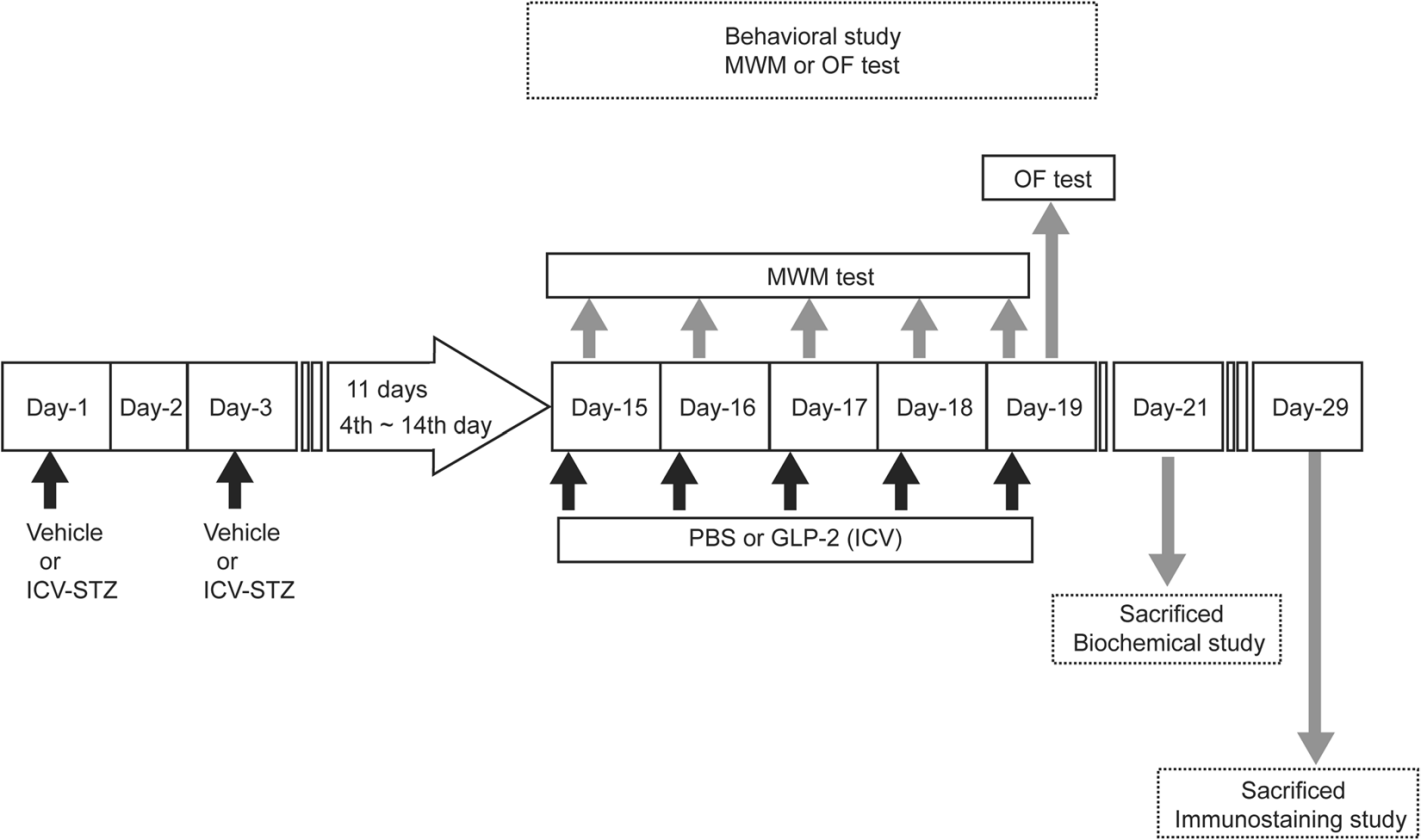
A schematic image of the time scale of the experimental design.

The experimental schedule is shown in Fig. 1. A total of 89 ddY mice were used in the experiments. Mice were divided into three groups (29, 29, 31, respectively), which were subjected to (1) the MWM test, (2) open-field (OF) test and then sacrificed for the immunostaining test, or (3) biochemical test. Moreover, mice were randomly divided into four groups (Control + PBS, Control + GLP-2, ICV-STZ + PBS, ICV-STZ + GLP-2). The control groups were subjected to the bilateral intracerebroventricular (ICV) administration of PBS (STZ vehicle, on Days-1and -3). Since we attempted to minimize the number of animals, mice used in the OF test were scarified on days 29 after the first ICV-STZ administration for the immunostaining test.

### ICV-STZ-induced dementia

The ICV administration (5 μl/ventricle) was performed according to the previously reported procedures^6,21^ using a 50-μl Hamilton microsyringe with a 28-gauge needle under brief isoflurane anesthesia. Two doses of vehicle or STZ (3 mg/kg, ICV) were administered bilaterally on Days-1 and -3 according to the procedure of Sodhi and Singh^22^. STZ was dissolved in PBS solution, which was made freshly just before administration.

### GLP-2 administration

GLP-2 was dissolved in PBS. Since the ability of GLP-2 to penetrate the blood-brain barrier remains unknown, GLP-2 (0.6 μg) or PBS (vehicle control) was administered to both lateral ventricular regions of the mouse brain. GLP-2 was administered 30 min before the MWM test once a day for 5 days (Day-15–19). The earlier studies have been shown that a treatment period of 4–6 days resulted in a significant increase in small intestinal weight, crypt/villus lengthening, and increased crypt cell proliferation rates using mouse and rat models^23–25^, indicating that the GLP-2 administration once daily for several days was necessary for cell proliferation. GLP-2 was thus administered for 5 days in this study.

### Behavioral test

#### MWM test

The MWM test was started on the Day-15 (Fig. 1) and was performed as described previously with some modifications^26^. A circular pool (147 cm in diameter, 45 cm in height) was filled to a depth of 25 cm with clear water (25 ± 1 °C). A clear platform (12 cm in diameter) was submerged 1 cm below the water surface. The platform was located in the center of the southwest quadrant. Mice were given three training trials each day for 5 consecutive days. In each training trial, mice were placed in the water facing the pool wall at one of four randomly selected starting locations (north, south, east, west). Latency to reach the platform was recorded up to 120 s. When the mouse found and got onto the platform, it was allowed to stay on the platform for 10 s. If the mouse failed to reach the platform within 120 s, it was guided to it and left there for 10 s. After each training trial, the mouse was dried with a towel and allowed to remain in a cage for an inter-trial interval of 15 min. The escape latency needed by mice to find the platform (in seconds) was measured using computer software (Smart Junior; Panlab SL, Barcelona, Spain).

#### OF test

Eighteen days after the first ICV-STZ administration, the OF test was performed as described previously with some modifications^6^. The OF apparatus consisted of a square area (40 × 40 cm) with 25-cm-high opaque walls. The floor was divided into 16 equal squares by lines. The central area comprised the central 4 squares (20 × 20 cm). Mice were placed in a corner of the OF facing the opaque walls. The number of crossings in the central area and the total number of crossings were then counted for 5 min. The apparatus was wiped down with paper after the removal of each animal.

### Biochemical parameters

Hippocampal tissue from these mice was homogenized in phosphate buffer (pH 7.4) on Day-21 because maximum changes in the thiobarbituric acid reactive species (TBARS) and glutathione (GSH) levels have been reported to occur 21 days after the first ICV-STZ administration^12^ by the protocol design based on the earlier study^27^.

### Estimation of TBARS levels

TBARS levels were measured according to the method of Ohkawa *et al*.^28^ with slight modifications using 1, 1, 3, 3-tetraethoxypropane as the standard. Three hundred microliters of 30% trichloroacetic acid (TCA), 150 μl of 5 N HCl and 300 μl of 2% w/v 2-thiobarbituric acid (TBA) were added to 500 μl of tissue homogenate in phosphate buffer (pH 7.4), and the mixture was heated at 90 °C for 15 min. The mixture was centrifuged 12,000 × *g* at 4 °C for 10 min. A pink colored supernatant was obtained, which was measured spectrophotometrically at 532 nm.

### Estimation of reduced GSH levels

The GSH levels were measured according to the method of Beutler *et al*.^29^ with slight modifications. The tissue homogenate was mixed with an equal amount of 10% TCA and centrifuged at 2,000 × *g* at 4 °C for 10 min. The supernatant was used to estimate GSH levels. A total of 2.5 ml phosphate buffer (pH 8.0) and 0.5 mg of DTNB were added to 0.25 ml of processed tissue sample, and the mixture was shaken vigorously with a vortex. Absorbance was read at 412 nm within 15 min.

### Histology

Mice were anesthetized with sodium pentobarbital (50 mg/kg) and transcardially perfused with 0.1 M phosphate buffer (pH 7.4), followed by 50–100 ml of 3% formaldehyde 120 min on days 29 after the first ICV-STZ administration by the protocol design based on the earlier studies^17,30^. The brain was removed, postfixed in the same fixative solution for 24 h at 4 °C, and cryoprotected by immersion in 30% (w/v) sucrose in PBS at 4 °C until the block sank^31^. Frozen tissues were sectioned on a cryostat (CM1560S; Leica Microsystems, Wetzlar, Germany) into coronal 20-μm-thick sections for the immunohistochemical staining of the proliferative marker, Ki67, and the immature neuron marker, doublecortin (DCX). Free-floating immunohistochemistry for Ki67 and DCX was performed, the first step of which was antigen retrieval. Sections were incubated with 0.3% hydrogen peroxide in methanol for 30 min, and then in normal goat serum (Millipore) with 0.3% Triton X-100 in PBS, followed by an overnight incubation at 4 °C in rabbit anti-Ki67 (1:1000, Ab-4, Thermo Fisher Scientific, Fremont, CA, USA) or goat anti-DCX (1:200, sc-8066; Santa Cruz, CA, USA). The sections were then incubated with biotinylated goat anti-rabbit IgG (1:200; Vector Laboratories Inc, Burlingame, CA, USA) or biotinylated goat anti-goat IgG (1:500; Vector) for 2 h. The sections were further incubated in ABC reagent (1:100; Vector Elite ABC Kit) for 90 min at room temperature. The reaction was revealed by 0.025% DAB with 0.01% hydrogen peroxide and then counterstained with Nissl, as described previously^31^.

We stained 20 sections per animal. To quantitatively analyze the number of Ki67- or DCX-positive cells in the subgranular zone (SGZ) of the hippocampal dentate gyrus (DG), 20 sections at 20-μm intervals were selected from each animal according to anatomical landmarks corresponding to Bregma −1.34 mm of the mouse brain atlas^32^.

### Data and statistical analyses

All values are given as means ± SEM. The significance of differences was evaluated using a parametric one-way or two-way analysis of variance (ANOVA) followed by Bonferroni’s multiple comparison test. In all cases, significance was set at *P* < 0.05. Statistical analyses were performed using Graphpad Prism 7 (Graphpad Software, San Diego, CA, USA).

### Ethics statement

All experimental protocols were approved by the Institutional Animal Care and Use Committee at Tokyo University of Science, and were conducted according to the guidelines of the National Institute of Health and the Japan Neuroscience Society.

## Results

### Effects of the administration of GLP-2 on ICV-STZ induced memory impairments in the MWM test

**Figure 2:**
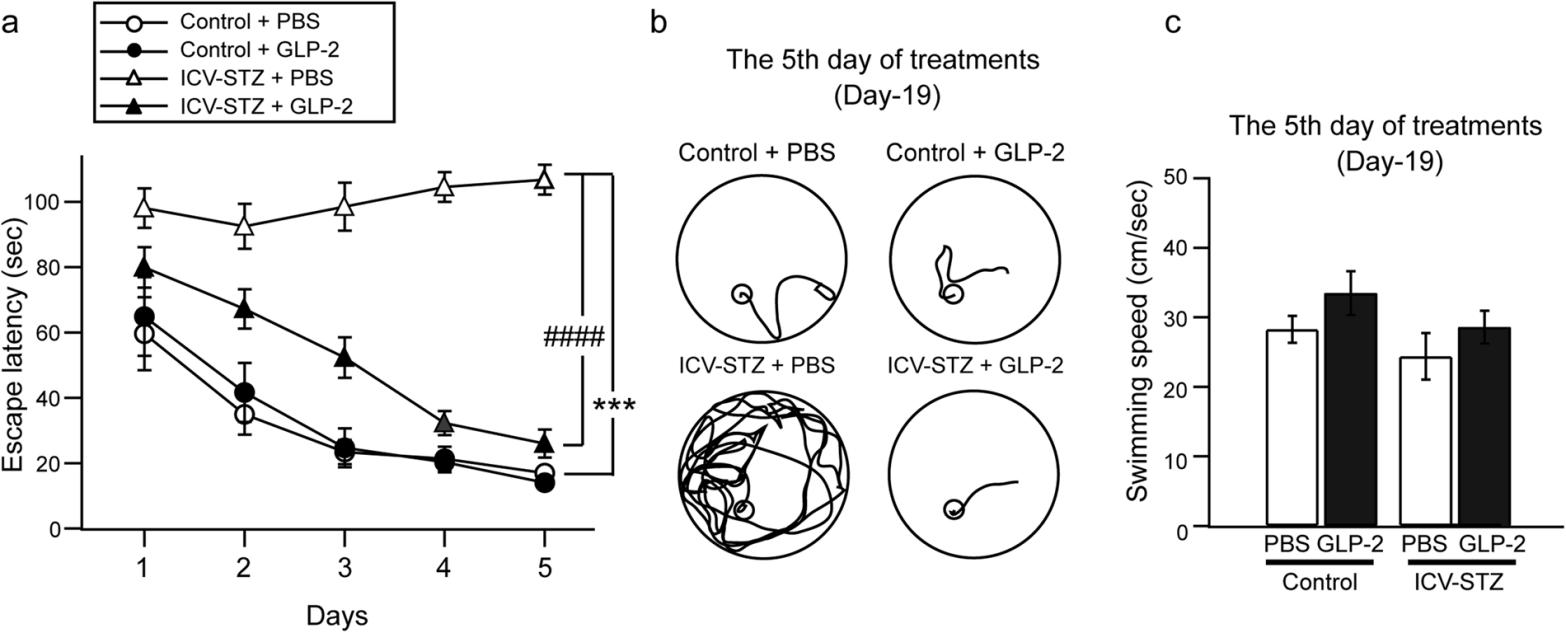
Effects of GLP-2 on spatial learning memory in the MWM test. Mice were tested for 5 consecutive days. (**a**) Time before reaching the hidden platform during five days of training. ***P < 0.001 vs. the control + PBS group, ^###^*P* < 0.001 vs. the ICV-STZ + PBS group (two-way repeated ANOVA). (**b**) Representative traces of the acquisition of spatial memory with the hidden platform on Day-19. (**c**) The total distance was recorded over 2 min on Day-19. ***P < 0.001 vs. the control + PBS group, ^###^*P* < 0.001 vs. the ICV-STZ + PBS group (one-way ANOVA). Results are expressed as means ± SEM (n = 6–9).

We examined hippocampus-dependent memory using the MWM test on Days-15 to -19. The latency to find the platform (escape latency) and swimming speed were shown in Fig. 2a,c, respectively. The escape latency of the control mice + PBS group decreased gradually during the 5 days of training. However, the ICV-STZ + PBS group (106.7 ± 4.6 s, *n* = 8) took a significantly longer time to find the platform than that of the control + PBS group on the 5th day of training (17.0 ± 1.2 s, *n* = 6) ([F(4, 60) = 12.99, ***P < 0.001]). The administration of GLP-2 (0.6 μg/day, ICV) for 5 days to the ICV-STZ + GLP-2 group resulted in a significantly shorter time to find the platform (26.0 ± 4.3 s, *n* = 9) than that of the ICV-STZ + PBS group (106.7 ± 4.6 s, *n* = 8) [F(4, 48) = 6.06, ^###^*P* < 0.001]. Swimming speed did not significantly differ among the groups (*P* > 0.05) (Fig. 2c). These results indicate that GLP-2 improved spatial learning impairments in ICV-STZ-treated mice without inducing motor deficits.

### Effects of the administration of GLP-2 on OF behaviors in ICV-STZ-treated mice

**Figure 3:**
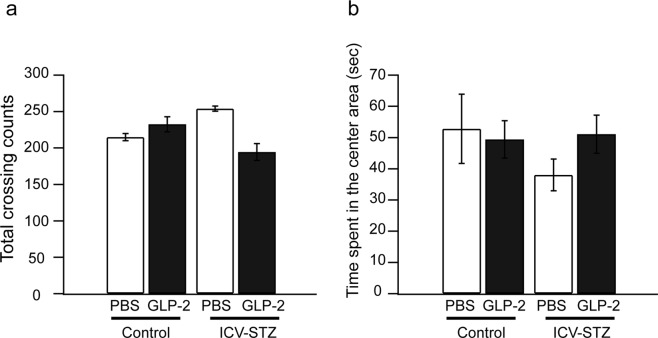
Effects of GLP-2 on open-field behaviors in ICV-STZ-treated mice. The total number of crossings (**a**) and time spent in the central area (**b**) were assessed the fifth day of the ICV administration of GLP-2 or PBS. Results are expressed as means ± SEM (n = 5–9).

We performed the OF test to investigate the effects of GLP-2 on locomotion activity and emotional responses 19 days after the first ICV-STZ administration. As shown in Fig. 3, GLP-2 (ICV) had no effect on the total number of crossings (*P* > 0.05) or the number of crossings in the central area (*P* > 0.05).

### Estimation of parameters of oxidative stress in the hippocampus of ICV-STZ-treated mice

**Figure 4:**
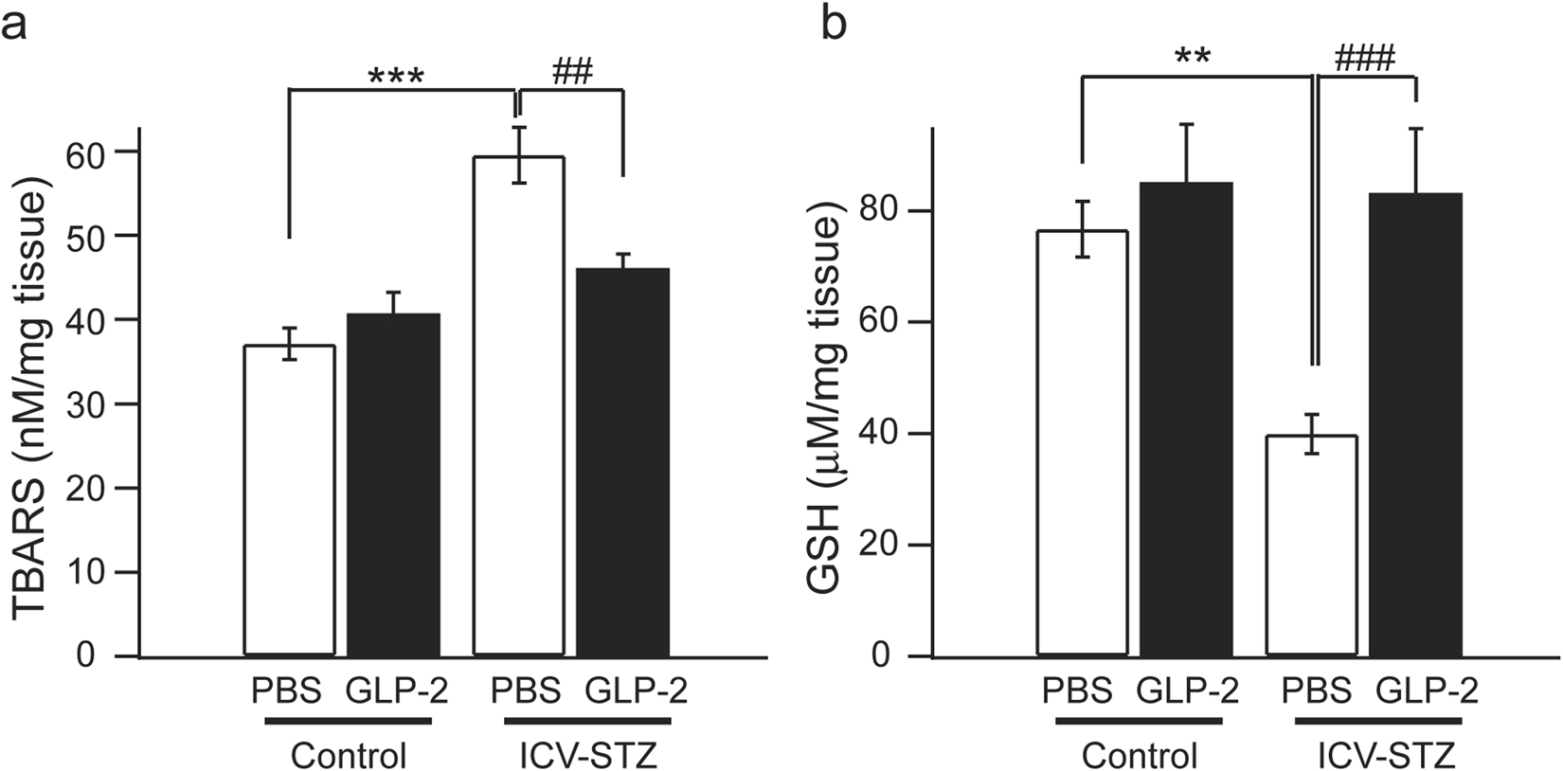
Effects of GLP-2 on hippocampal TBARS and GSH in ICV-STZ-treated mice. The restoration of ICV-STZ-induced increases in TBARS levels (**a**) and decreases in GSH levels (**b**) by GLP-2 was significantly better than that by PBS. Results are expressed as means ± SEM (n = 6–10). **P < 0.01, ***P < 0.001 vs. the control + PBS group, ^##^*P* < 0.01, ^###^*P* < 0.001 vs. the ICV-STZ + PBS group (one-way ANOVA).

TBARS and GSH levels were measured in the hippocampus 20 days after the first ICV-STZ administration. As shown in Fig. 4, TBARS levels were significantly higher and GSH levels were significantly lower in ICV-STZ-treated mice than in control mice (TBARS ***P < 0.001, GSH **P < 0.01). The administration of GLP-2 (0.6 μg/day, ICV) for 5 days significantly prevented ICV-STZ-induced changes in TBARS levels (^##^*P* < 0.01) and GSH levels (^###^*P* < 0.001).

### Effects of the administration of GLP-2 on the number of Ki67-positive or DCX-positive cells in ICV-STZ-treated mice

**Figure 5:**
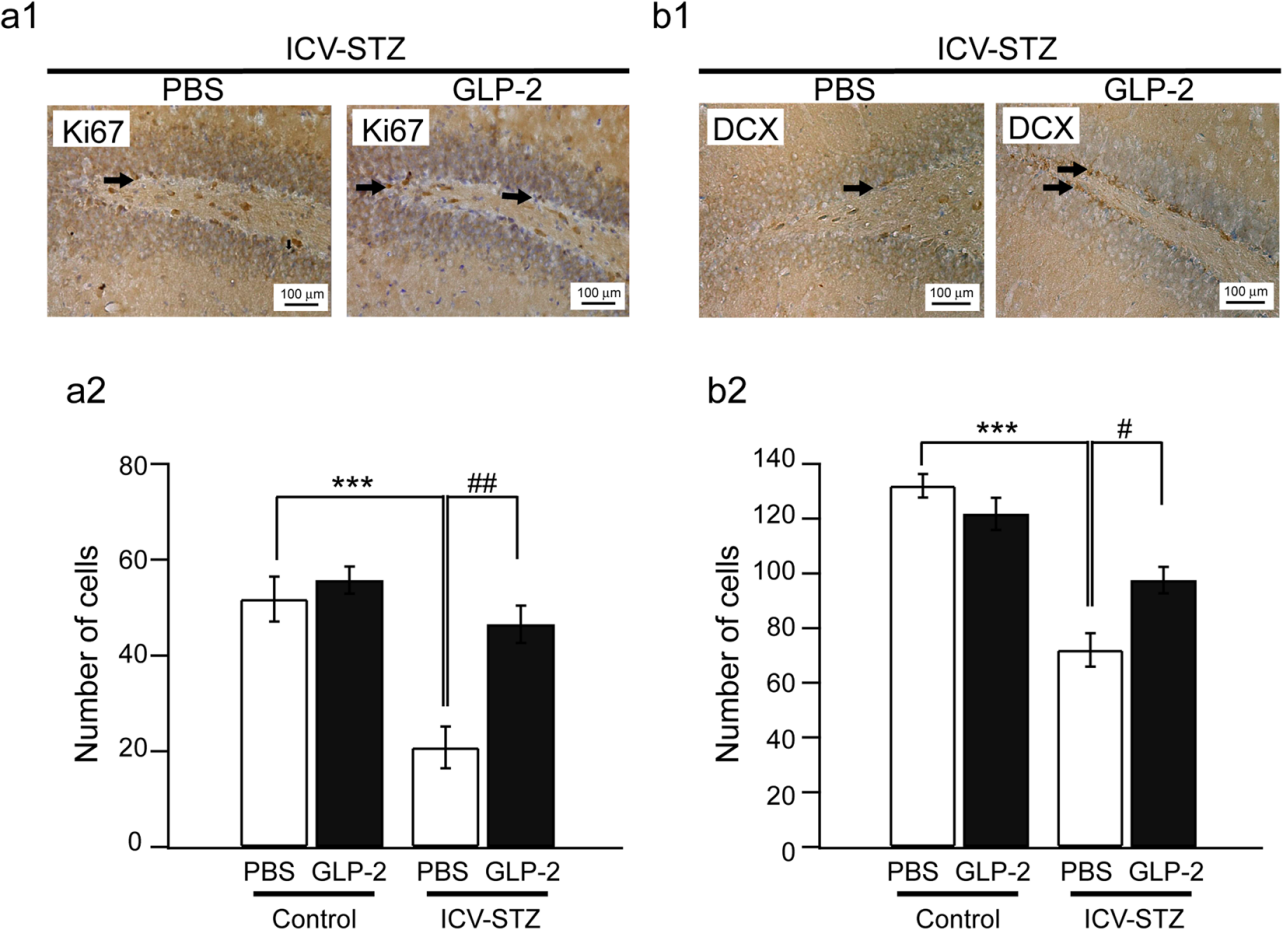
Effects of GLP-2 on the number of Ki67-positive or DCX-positive cells in ICV-STZ-treated mice. The Immunohistochemistry for Ki67 or DCX in the SGZ of the hippocampal DG. A typical photomicrograph shows Ki67-positive (a1) or DCX-positive (b1) cells in the SGZ of ICV-STZ + PBS or + GLP-2-treated mice. A filled arrow denotes an example of Ki67-positive or DCX-positive cells. Bar graphs show the mean numbers of Ki67-positive (a2) or DCX-positive (b2) cells. Results are expressed as means ± SEM (n = 4). ***P < 0.001 vs. the control + PBS group, ^#^*P* < 0.05, ^##^*P* < 0.01 vs. the ICV-STZ + PBS group (one-way ANOVA).

We investigated the protective activities of GLP-2 against impaired neurogenesis in ICV-STZ-treated mice. Cellular proliferation in the SGZ of the hippocampal DG was examined by counting the number of Ki67-positive cells in ICV-STZ-treated mice. The phenotypic distribution is shown in Fig. 5a1. In ICV-STZ-treated mice, the number of Ki67-positive cells in the SGZ was significantly higher after the administration of GLP-2 than after the administration of PBS (Fig. 5a2) (*n* = 4, ^##^*P* < 0.01). Immature neurons in the SGZ were counted based on DCX immunohistochemistry. The phenotypic distribution is shown in Fig. 5b1. In ICV-STZ-treated mice, the number of DCX-positive cells in the SGZ was significantly higher after the administration of GLP-2 than after the administration of PBS (Fig. 5b2) (*n* = 4, ^#^*P* < 0.05).

## Discussion

In the present study, we investigated the potential of GLP-2 in an animal model of sporadic AD induced by the ICV administration of STZ. In ICV-STZ-treated mice, we demonstrated the memory restorative effects of GLP-2 in the MWM test. The MWM test employed in the present study is one of the most accepted models to evaluate spatial learning and memory in rodents^33^. The hippocampus is an important brain area for learning and memory^34^. We showed that ICV-STZ significantly promoted oxidative stress, such as decreases in GSH levels and increases in TBARS levels in the hippocampus, which were consistent with previous findings obtained in the whole brain^35,36^. We also found that GLP-2 restored these ICV-STZ-induced biochemical alterations. Since the ICV-STZ model is considered to be predominately based on free radical generation^37^, we suggest that GLP-2 improves memory functions in ICV-STZ-treated mice through changes in antioxidant activity. In addition to a previous study showing that GLP-2 exerted antioxidant effects against TNF-α/actinomycin D-induced small intestinal damage^38^, we herein demonstrated for the first time the antioxidant effects of GLP-2 in the CNS. Although it is not clear whether the effect of GLP-2 disappears in the course of time or is persistent, GLP-2 probably inhibited the ongoing oxidative stress sequences in the hippocampus induced by ICV-STZ during the repeated administration of GLP-2. However, further studies are needed in order to elucidate the duration of the effects of GLP-2 and the underlying mechanisms in more detail.

The ICV administration of STZ has been reported to produce similar characteristic pathology of sporadic AD such as altered glucose metabolism, oxidative stress, synaptic dysfunction, protein kinases such as protein kinase B/C, glycogen synthase-synthase-3β (GSK-3β) activation, tau hyperphosphorylation, Aβ deposition of Akt/PKB, insulin receptor (IR) signaling molecule, and insulin resistance in brain^39^. Oxidative stress appears to be one of the most important factors for triggering cellular death in AD and the mediator of several processes contributing to AD pathophysiology^40^. Earlier studies showed that the ICV administration of STZ also caused oxidative stress commonly decreases in GSH levels and increases in TBARS levels^35,36^, which were consistent with this study. Since GLP-2 significantly restored these ICV-STZ-induced changes, further studies are needed in order to elucidate the mechanisms underlying the antioxidative effects of GLP-2 in more detail.

Oxidative stress has been reported to stimulate such as lipid peroxidation, DNA damage, cell death, the inhibition of cell growth, and the inhibition of cell proliferation^34^, and then we also investigated the effects of GLP-2 on neurogenesis in ICV-STZ-treated mice. The SGZ of the hippocampal DG is known to be one of the brain areas at which neurogenesis continues throughout life in adult mammals, including humans^41,42^. Extensive evidence has suggested that new neurons generated through this process play a crucial role in hippocampus-dependent learning and memory (e.g., spatial memory and orientation) and recovery from neuronal injury^43^. The present results showing that the ICV administration of STZ reduced neurogenesis in the SGZ were consistent with previous findings^16,17^, and GLP-2 was herein shown to have the ability to restore neurogenesis in ICV-STZ-treated mice. Taken together with oxidative stress affecting cell proliferation and growth^40^, we suggest that GLP-2 reduces ICV-STZ-induced oxidative stress, resulting in protective effects on neurogenesis. Since a recent study showed that the treatment of a novel GLP-1/GIP dual receptor agonist for 2 weeks induced insulin signaling re-sensitized as evidenced by a reduction of phospho-insulin receptor substrate-1 (IRS-1)^ser1101^ levels and phospho-Akt^ser473^ up-regulation in ICV-STZ-treated rats^44^, we would examine whether the treatment of GLP-2 affect glucose metabolism in future.

We previously reported that GLP-2 exerts antidepressant-like effects and enhances neurogenesis in adrenocorticotropic hormone-treated mice, which have been considered to be an animal model of tricyclic antidepressant-resistant depression^31^. Although cognitive dysfunction is the main symptom of AD, considerable patients with AD have been reported to suffer from major depression^45,46^. We thus need to clarify whether GLP-2 exerts antidepressant-like effects in AD models in order to develop more efficient drugs.

## Conclusion

We herein demonstrated that GLP-2 significantly restored ICV-STZ-induced memory impairments as well as oxidative stress and neurogenesis deficits, and accordingly, propose that the memory restorative ability of GLP-2 is due to its potential to reduce oxidative stress.
